# The Solidification Behavior of AA2618 Aluminum Alloy and the Influence of Cooling Rate

**DOI:** 10.3390/ma7127875

**Published:** 2014-12-09

**Authors:** Yulin Liu, Ming Liu, Lei Luo, Jijie Wang, Chunzhong Liu

**Affiliations:** Liaoning Provincial Key Laboratory of Light Alloys and Processing Technology, School of Materials Science and Engineering, Shenyang Aerospace University, 37 Daoyi Avenue S., Shenyang 110136, China; E-Mails: liuming.sau@gmail.com (M.L.); lluo.sau@gmail.com (L.L.); wangjijie@sau.edu.cn (J.W.); czliu@sau.edu.cn (C.L.)

**Keywords:** aluminum alloy, AA2618 alloy, solidification behavior, near-rapid cooling, microstructure

## Abstract

In AA2618 aluminum alloy, the iron- and nickel-rich intermetallics formed during solidification are of great effect on the mechanical properties of the alloy at both room temperature and elevated temperatures. However, the solidification behavior of the alloy and the formation mechanism of the intermetallics during solidification of the alloy are not clear. This research fills the gap and contributes to understanding the intermetallic of the alloy. The results showed that cooling rate was of great influence on the formation of the intermetallics. Under the condition of slow cooling, the as-cast microstructures of the alloy were complex with many coarse eutectic compounds including Al_9_FeNi, Al_7_(CuNi)_5_, Si, Al_2_Cu and Al_2_CuMg. The phase Al_9_FeNi was the dominant intermetallic compound, which precipitated at the earlier stage of the solidification by eutectic reaction L → α-Al + Al_9_FeNi. Increasing the cooling rate would suppress the formation of the coarse eutectic intermetallics. Under the condition of near-rapid cooling, the as-cast microstructures of the alloy consisted of metastable intermetallics Al_9_FeNi and Al_2_Cu; the equilibrium eutectic compounds were suppressed. This research concluded that intermetallics could be refined to a great extent by near-rapid cooling.

## 1. Introduction

AA2618 alloy is an Al–Cu–Mg system alloy. With the addition of iron and nickel, it is suitable for applications at elevated temperatures. In most aluminum alloys, iron is considered a harmful impurity element, due to the formation of iron-rich intermetallics, which deteriorate the mechanical properties of alloys. However, in AA2618 aluminum alloy, iron is an important alloying element. It combines with nickel to form an insoluble iron- and nickel-rich intermetallic Al_9_FeNi. The size, morphology and distribution of the intermetallic in the alloy are of great influence on the mechanical properties of the alloy at both room temperature and elevated temperatures. The fine dispersoid particles play a positive role in improving the mechanical properties of the alloy at elevated temperatures by enhancing the microstructural stability of the alloy under thermal exposure [1,2]. However, as more iron and nickel are added to the alloy, more coarse iron- and nickel-rich intermetallics will form. The coarse iron- and nickel-rich intermetallics will act as stress risers and deteriorate the mechanical properties of the alloy. The extent of the deterioration depends on the size, shape and type of the intermetallics. The platelet-like iron-rich phases have usually been considered most detrimental to the mechanical properties of aluminum alloys due to their brittle features and the stress concentration caused by the needle-like morphology [3,4]. Therefore, efforts need to be dedicated to developing means of controlling the precipitation, growth and morphology of these iron-rich intermetallic phases during solidification. However, so far, the solidification behavior of AA2618 alloy has not been studied and the formation mechanism of its intermetallic compounds during solidification of the alloy is not clear.

Over the years, lots of research has been done to improve alloy AA2618. For example, Özbek studied heat treatment [5]; Cavaliere investigated hot and warm formation [6]; Sakthivel *et al.* studied reinforcing by SiC particles [7]; Yu *et al*. studied the effect of Al3(Sc, Zr) phases [8]; Oguocha and Yannacopoulos investigated natural ageing behavior [9]; Wang *et al*. investigated the influence of deformation ageing treatment [10]; Novy *et al*. studied the microstructure change during aging [11]; Wang *et al.* investigated the microstructural evolution during creep [12]. All these researches on AA2618 aluminum alloy were about the microstructure and mechanical properties of the alloy. No research on the solidification behavior of AA2618 alloy and the formation and control of the intermetallics in the alloy was reported. A systematical study is necessary to resolve these questions.

As a first step, it is necessary to identify and understand the factors that will affect the solidification behavior and the as-cast microstructure of commercial alloys, especially, the formation and refinement of the iron-rich intermetallics. Recently, Liu *et al*. [4,13,14,15] made systematical investigations into the formation of the iron-rich intermetallics in Al–Cu 206 cast alloys. It was found that the formation of the iron-rich intermetallics was greatly affected by the alloy’s chemical composition and its cooling conditions. AA2618 aluminum alloy is much more complicated than Al–Cu 206 alloy in terms of chemical composition. It can therefore be assumed that the solidification behavior of AA2618 alloy and the formation mechanism of the intermetallics in the alloy are also very complicated.

Cooling rate is an important factor that would affect the solidification behavior of AA2618 alloy. It is hypothesized that under the condition of near-rapid cooling, the iron-rich intermetallic compounds formed during the solidification of the alloy could be greatly refined, minimizing the formation of coarse intermetallics. Should this hypothesis hold true, under near-rapid cooling, coarse intermetallics could be avoided when more alloying elements are added, which would improve the mechanical properties of the alloy. The cooling of the continuous strip casting process of aluminum alloy is a near-rapid cooling with a cooling rate of 10^1^–10^2^ K·s^−1^. New material with high alloying elements contents could thus be developed and produced by the continuous strip casting process. Therefore, it is of theoretic and commercial interesting to conduct a research on the solidification behavior of AA2618 alloy under the condition of near-rapid cooling.

In this paper, the research was focused on the AA2618 alloy. Systematic research on the solidification behavior of the alloy, the formation of intermetallic Al9FeNi and the influence of cooling rate will be helpful in refining the coarse intermetallics, in return, improving the mechanical properties of the alloy and optimizing the alloy composition.

## 2. Experimental Procedures

The material used in this study was a commercially produced thick plate, with a thickness of 20 mm, in T6 temper. Its composition was analyzed by emission spectroscopy and the result was as follows, in wt%: Al–2.21 Cu–1.29 Mg–1.10 Fe–1.03 Ni. Other elements included 0.17 Si, 0.03 Mn, 0.07 Zn and 0.09 Ti. The samples used for remelting were cut from the plate. The investigations were carried out by remelting different samples of the alloy and solidifying them at different cooling rates, and then analyzing the as-cast microstructures of each sample by metallographic examination with optical microscopy (OM) and electronic scanning microscopy (SEM) with Energy Dispersive X-ray Detector (EDX).

For each remelting-solidification test, approximately 80 g of material was remelted in an alumina ceramic crucible in an air furnace by heating the material to 1023 K (750 °C). The molten materials were kept for at least 30 min at the aforementioned temperature, in order to melt the material entirely and to homogenize the composition. The molten materials were then solidified under the different conditions summarized below in Table 1. In these experiments, the temperature was measured using a K-type thermocouple. The thermocouple was inserted along the centerline of the crucible or mold halfway into the melt. An evolution of temperature with time was recorded every 0.1 second. The average cooling rate of each sample was calculated by using the formula d*T*/d*t* and was computed from the approximate straight-line portion of the cooling curve before the start of the solidification. Labsys Evo DSC (differential scanning calorimeter)-1600 from Setaram Instrumentation (Caluire, France) was used to analyze the heat flow during solidification.

**Table 1:** Summary of cooling conditions.

Sample ID	Cooling condition	Cooling rate, K·s^−1^
FC (Furnace cooling)	Cooled in crucible in furnace	0.037
AC (Air cooling)	Cooled in crucible in air	1.47
IC (Iron mold cooling)	Cast in iron mold, casting size: Φ 25 mm × 50 mm	39.5
WC (Water cold mold cooling	Cast in a double-side water cooled iron mold, casting size: 50 mm × 40 mm × 14 mm	88.2

The solidification sequences of the alloy were revealed by using an interrupted water quenching method—interrupting the solidification process and quenching the solidifying sample into water. This method was previously used by the present authors [16] and recently by Liu *et al*. [15] and has been proven to work. By quenching samples at different temperatures and by distinguishing the solidified structure and the water-quenched structure of the samples, the solidification process of the alloy could be revealed and the solidification sequence and microstructure could be determined. During solidification, the temperatures of the solidifying samples were in-line monitored. Once the pre-determined temperature (or time) was reached, the samples were quenched into water immediately. The transit time of the sample from furnace to water was less than one second.

Samples for metallographic investigations were cut from the solidified ingots in the vicinity of the thermocouple’s tip, ground with SiC paper, polished with 3 µm alumina water solution and finally polished using diamond solution. The samples were etched with a reagent (25 vol% HNO_3_ + 2 vol% HF + 73 vol% H_2_O) and the microstructures of the samples were examined using an OLYMPUS GX71 optical microscope from Olympus Corporation (Shinjuku, Japan), A Zeiss scanning electron microscopy with energy dispersive X-ray (EDX) analyzer from Zeiss Group (Oberkochen, Germany) and a Shimdzu electronic probe microanalyzer (EPMA-1610) from Shimdzu Corporation (Kyoto, Japan) were employed to identify the intermetallic compounds.

## 3. Results and Discussion

### 3.1. Microstructure Characteristics and Solidification Sequence of the Alloy under Slowing Cooling (Samples FC and AC)

#### 3.1.1. Microstructure Characteristics

**Figure 1:**
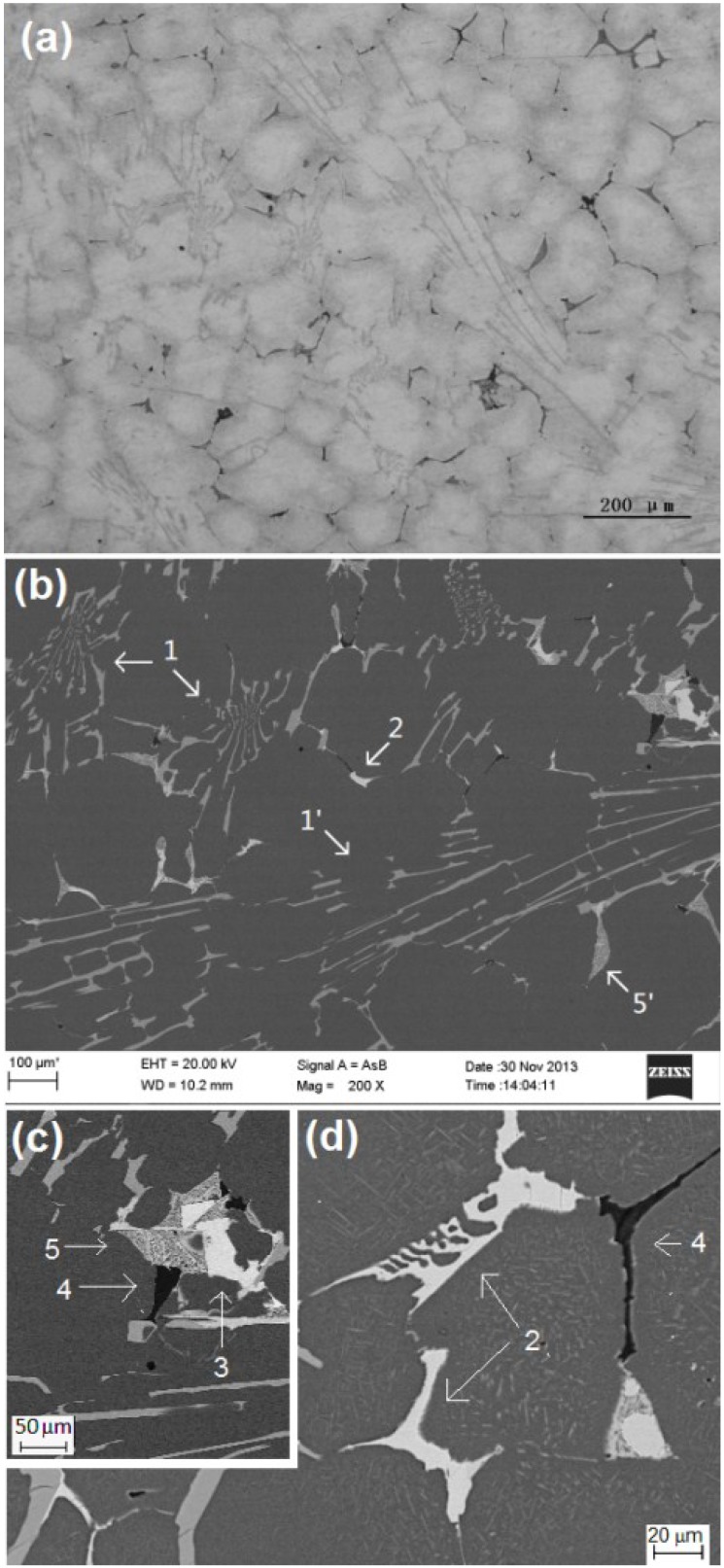
The as-cast microstructures of Sample FC (**a**) OM image; (**b**)–(**d**) SEM backscatted electron images.

**Figure 2:**
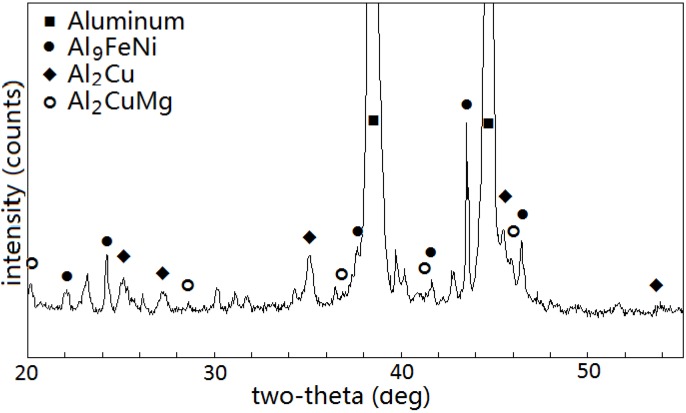
X-ray diffraction spectra of the Sample FC.

The as-cast microstructures of Sample FC solidified at a cooling rate of 0.037 K·s^−1^ were revealed by OM and SEM, as shown in Figure 1. They consisted of primary α-Al matrix and lots of intermetallic compounds. As marked in the backscatted electron images of the microstructures in Figure 1b–d, at least five intermetallic phases could be identified: flower-like eutectic cluster in dark gray (marked as 1), flake/plate-like phase in light gray (marked as 2), blocky phase in light gray (marked as 3), black phase (marked as 4) and eutectic cellular in dark gray (marked as 5). The XRD (X-ray diffraction) patterns of the sample revealed the presence of Al_9_FeNi, Al_2_Cu and Al_2_CuMg, as shown in Figure 2.

A method for identifying intermetallics was to measure the chemical composition of the phases using EDX and EPMA. The results of the EDX examinations are summarized in Table 2. At least five spots were measured for each type of microstructures and no significant difference among the measurements was found. The EPMA results are also summarized in Table 2. It was discovered that the findings from the two instruments were quite close. The chemical compositions of type 1 and type 3 microstructures well matched the stoichiometric composition of Al_9_FeNi and Al_2_Cu, respectively. Therefore, it was certain that the flower-like eutectic cluster (1) was Al_9_FeNi and the blocky phase in light gray (3) was Al_2_Cu. The flake/plate-like phase (2) contained Al, Cu and Ni with an approximate ratio of Al_7_Cu_3_Ni_2_. According to the Al–Cu–Ni phase diagram [17], there were several types of closely-related structures of Al–Cu–Ni phases named as τ*_i_*. τ_7_ was a structure of Al_7_(CuNi)_5_. The composition of the flake/plate-like phase (2) well matched this structure. Therefore, it was reasonable to define the flake/plate-like phase (2) as Al_7_(CuNi)_5_. The dark gray eutectic cellular (5) contained Al, Cu and Mg with an approximate ratio of Al_5_Cu_3_Mg_2_. Since the eutectic compound was quite fine, the measurements might include some aluminum from the aluminum matrix, which caused the value of the measurements lower than their actual value in the phase. According to the Al–Cu–Mg phase diagram [18], at the aluminum corner, there were two eutectic compounds: Al_2_Cu (phase θ) and Al_2_CuMg (phase S). Although the composition of the dark gray eutectic cellular (5) deviated from Al_2_CuMg, considering the fact that phase Al_2_CuMg was the only phase that contained both Cu and Mg as solutes, it was reasonable to define the dark gray eutectic cellular (5) as Al_2_CuMg. The phase in black (4) contained mainly silicon. Although some aluminum was detected, it was reasonable to define this phase as silicon with other elements trapped within. The aluminum detected in the phase might have come from the aluminum matrix.

**Table 2:** Composition of intermetallic compounds in Sample FC measured by EDX and EPMA (at%).

Phase # as marked in Figure 1	EDX/EPMA	Al	Cu	Mg	Fe	Ni	Si	Suggested phase formula
1	EDX	81.35	0.51	-	8.84	9.30		Al_9_FeNi
	EPMA	81.52	0.47		8.06	9.74	0.22	
2	EDX	59.19	21.86	-	0.27	18.68		Al_7_(CuNi)_5_
	EPMA	59.47	22.49	0.02	0.11	17.89	0.02	
3	EDX	66.63	32.27	1.10	-	-	-	Al_2_Cu
	EPMA	65.67	32.71	1.22	0.01	0.32	0.05	
4	EDX	7.68	-	-	1.77		90.55	Silicon
	EPMA	1.95	0.39	2.13	0.01	0.06	95.47	
5	EDX	49.09	30.94	19.97	-	-	-	Al_2_CuMg
	EPMA	57.69	25.19	16.93	0.05	0.01	0.10	

**Figure 3:**
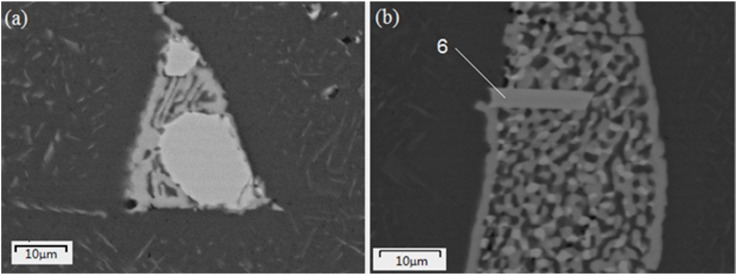
The morphologies of eutectic cellular: (**a**) eutectic α-Al + Al_2_CuMg; (**b**) eutectic α -Al + Al_2_Cu + Al_2_CuMg.

It was found by EDX and EPMA analyses that the composition of the radiated type structure, marked as 1' in Figure 1b, was almost the same as that of the flower-like structure, marked as 1. Therefore, it was assumed that both of the structures were intermetallic phase Al_9_FeNi. The Al_7_(CuNi)_5_ phase also displayed complex morphologies, such as lamellar or Chinese script type structure, as shown in Figure 1d. Two types of eutectic cellular were observed, marked as 5 and 5' in Figure 1b,c, which usually lie beside the blocky Al_2_Cu. The eutectic compound Al_2_CuMg formed a lamellar structure; Figure 3a shows a sample of this type of eutectic cellular. This type of eutectic cellular was more complex. It was a product of ternary eutectic reaction. The phase in light gray was Al_2_Cu, while the phase in dark gray was Al_2_CuMg. The presence of Mg made phase Al_2_CuMg display a little bit of darkness under the backscatted image. In addition, there was a plate-like (6) phase set in the eutectic cellular. EDX analysis indicated that it contained 70.21 at% Al, 6.16 at% Fe, 3.58 at% Ni and 20.05 at% Cu. It might be phase Al_7_Cu_2_Fe, as nickel was replaced by iron.

**Figure 4:**
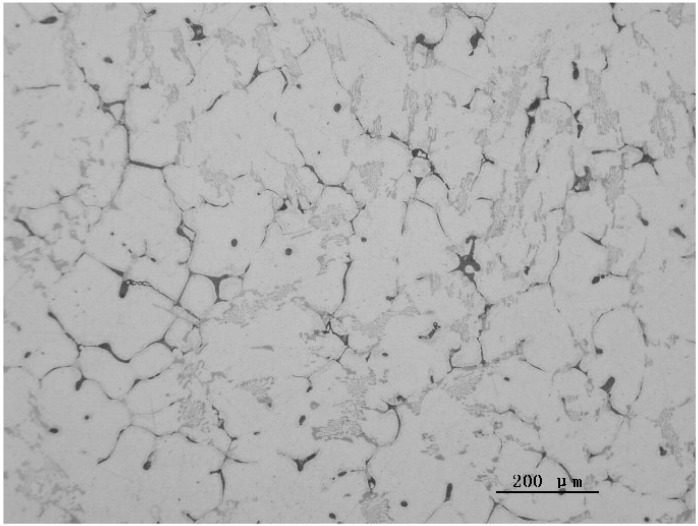
The as-cast microstructures of Sample AC.

Figure 4 shows the as-cast microstructure of Sample AC solidified at a cooling rate of 1.47 K·s^−1^. Some changes in microstructure were observed comparing to Sample FC. The eutectic cluster of Al_9_FeNi mainly displayed flower-like patterns; no more radiated type structure was observed. The eutectic cellular increased in quantity.

#### 3.1.2. Solidification Reaction and Sequence of the Alloy

**Figure 5:**
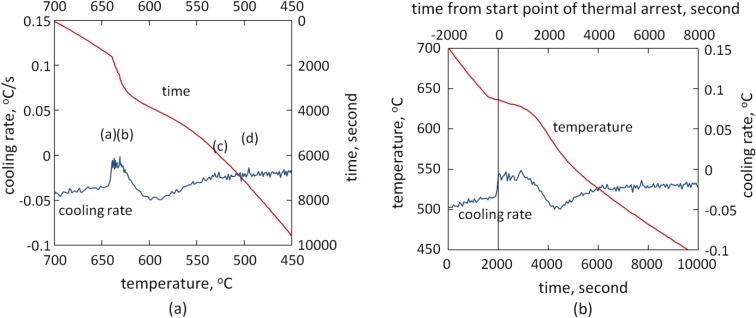
Thermal analysis curve and its first derivation of Sample FC: (**a**) cooling rate and cooling time *vs.* temperature; (**b**) temperature and cooling rate *vs*. cooling time.

Figure 5 shows the thermal analysis curve and its first derivative of Sample FC. Several peaks were observed, which were denoted as (a), (b), (c) and (d), corresponding to thermal events that occurred during solidification of the alloy. Combining the thermal analysis and the as-cast microstructures observed, it could be deduced that the thermal event associated with peak (a) corresponded to the formation of primary α-Al dendrites, which occurred at 912 K (639 °C); the other peaks were associated with the formations of the intermetallic phases.

**Figure 6:**
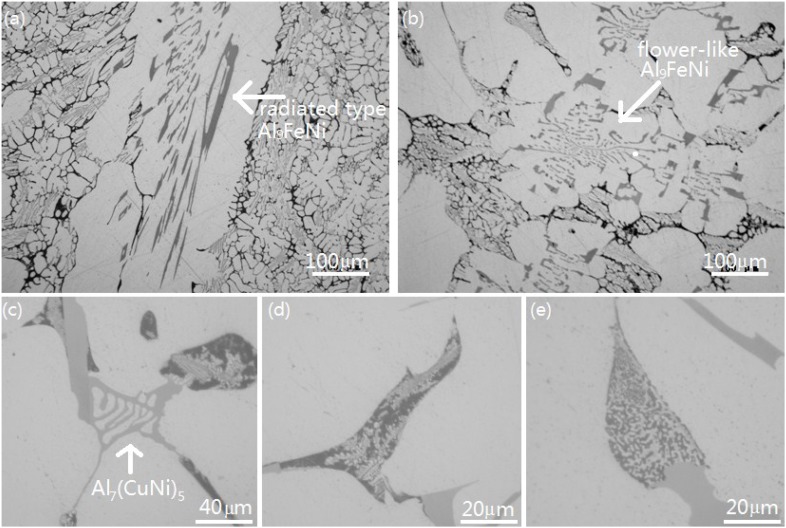
The microstructures after interrupted quenching at different temperatures: (**a**) 630 °C (16 min); (**b**) 628 °C (20 min); (**c**) 525 °C; (**d**) 495 °C and (**e**) 485 °C.

There were some peak-like fluctuations between peaks (a) and (b). In order to determine whether these peak-like fluctuations were associated with precipitation of any intermetallic and to link the peaks to real processes and reactions that happened during solidification, a series of interrupted water-quenchings were performed during the solidification of the alloy in order to study the intermetallics that form at different times and temperatures. At the beginning of solidification, the decrease in the temperature of the molten alloy was very slow. The first group of five interrupted water-quenchings was conducted every four minutes from the start of thermal arrest. The second group of interrupted water-quenchings was conducted at different temperatures during cooling: 873, 848, 798, 773 and 763 K (600, 575, 525, 500, and 490 °C). It was found that no precipitation of intermetallic occurred in the first three quenched samples. In other words, no intermetallic precipitated from the liquid after 12 min of solidification. In the fourth quenched sample, quenched after 16 min of solidification (at temperature of approximately 903 K (630 °C)), a large amount of long plate-like phases with a radiated type structure appeared, as shown in Figure 6a. The EDX analysis indicated that this phase possessed the chemical composition of Al_9_FeNi. These results confirmed that the peak-like fluctuations between peaks (a) and (b) were not associated with precipitation of any intermetallic. Al_9_FeNi was the first intermetallic that precipitated from liquid, which corresponded to peak (b) at 631.5 °C. The fraction of solid in this sample was about 28%. In the fifth sample, quenched after 20 min of solidification (at around 901 K (628 °C)), no new intermetallic was observed, but lots of Al_9_FeNi with flower-like morphology displayed within triangle grain boundaries (Figure 6b). The fraction of solid in this sample was about 75%.

In the samples that were quenched at 873 and 848 K (600 and 575 °C), the solid fraction increased to about 86% and 93%, respectively, but no new intermetallic was observed. Figure 6c shows that a new intermetallic, Al_7_(CuNi)_5_, was found in the sample quenched at 798 K (525 °C), which was slightly lower than the temperature of peak (c). In this sample, the solid fraction was about 96%.

**Figure 7:**
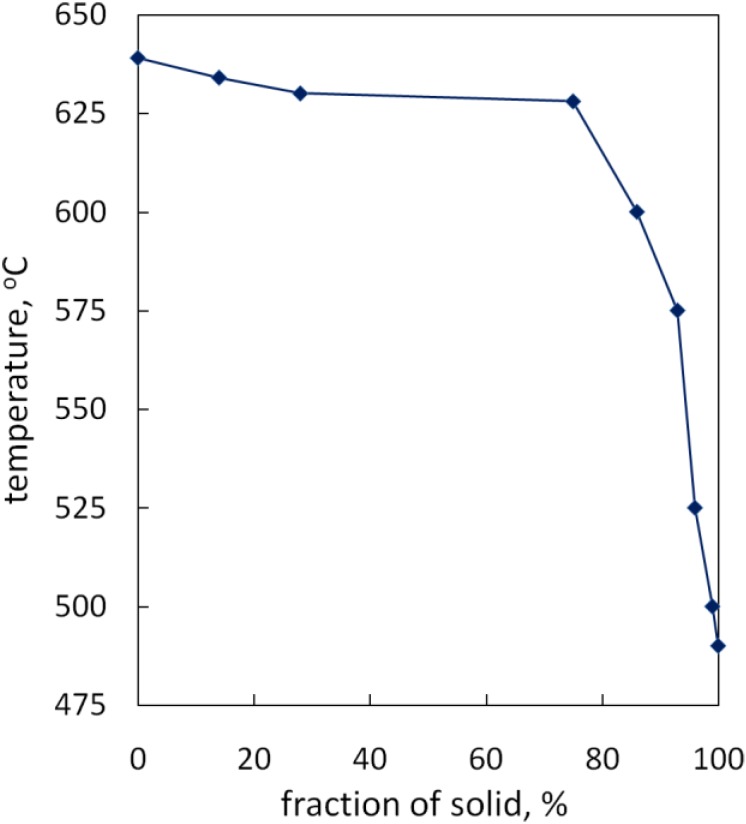
Solidification fraction *vs*. temperature.

In the sample that was quenched at 773 K (500 °C), the alloy had not yet fully solidified, some water-quenched structures could still be found, as shown in Figure 6d); the fraction of remaining liquid was about 1%. Fully solidified structure was observed in the sample quenched at 763 K (490 °C), see Figure 6e. It was certain that the solidification process terminated between 773 K (500 °C) and 763 K (490 °C). Figure 7 shows the solidification fractions *vs.* temperature.

**Figure 8:**
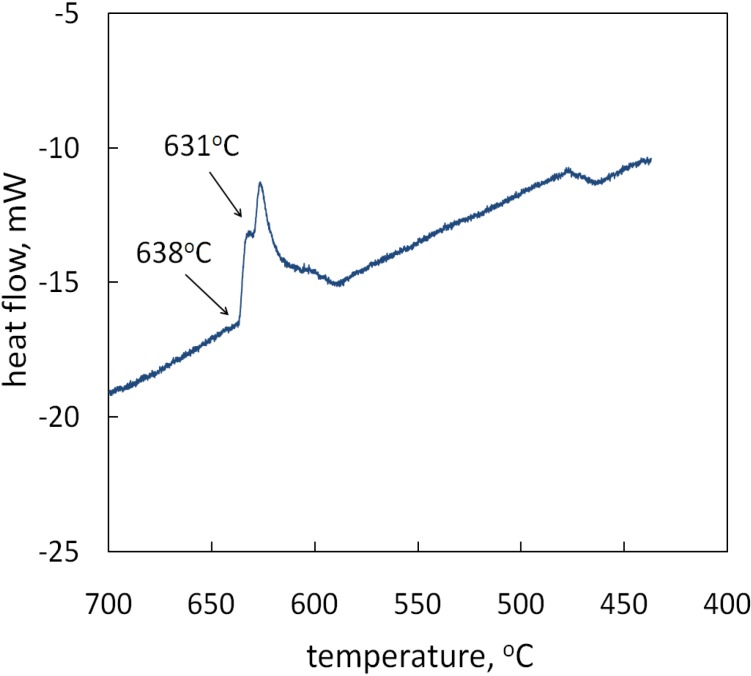
DSC curve of the alloy.

Setaram Labsys Evo DSC-1600 differential scanning calorimeter was used to analyze the heat flow during solidification. 10.0 mg material was heated to 710 °C, and then cooled to 430 °C at a cooling rate of 2 K·s^−1^, which was close to the cooling rate of Sample FC. The DSC curve is shown in Figure 8. Two reaction peaks were observed on the DSC curve at 638 and 631 °C. Upon cooling from 710 °C, the alloy began to solidify at 638 °C. A second solidification reaction was observed at 631 °C, which must have corresponded to the formation of intermetallic Al_9_FeNi. Since the sample for DSC analysis was very small (10.0 mg) and the quantities of other intermetallics (Al_7_(CuNi)_5_, Al_2_Cu, Al_2_CuMg) formed under slow cooling were also small, the reaction peaks for those intermetallics could be too small to be detected.

From the thermal analysis curve, phase diagrams of Al–Fe–Ni [19], Al–Cu–Ni [17] and Al–Cu–Mg [18], the as-cast microstructures observed, and the findings from the interrupted quenching tests, it could be assumed that α-Al + Si eutectic, α-Al + Al_2_Cu eutectic and/or α-Al + Al_2_Cu + Al_2_CuMg ternary eutectic formed in the final solidification zones. All possible solidification reactions and sequences were summarized in Table 3. The formation temperatures of primary α-Al and intermetallic Al_9_FeNi could be well defined. Reasonable ranges could be given to the formation temperatures of other intermetallics.

**Table 3:** Solidification sequence of Sample FC.

Peak	Solidification	Reaction	Temperature, K (°C)
(a)	Primary α-Al dendrite network	Primary	912–911 (639–638)
(b)	L → α-Al + Al_9_FeNi	eutectic	905–904 (632–631)
(c)	L → α-Al + Al_7_(CuNi)_5_	eutectic	Around 800 (527)
(d)	L → α-Al + Si	eutectic	773–763 (500–490)
(d)	L → α-Al + Al_2_Cu	eutectic	773–763 (500–490)
(d)	L → α-Al + Al_2_Cu + Al_2_CuMg	eutectic	773–763 (500–490)

**Figure 9:**
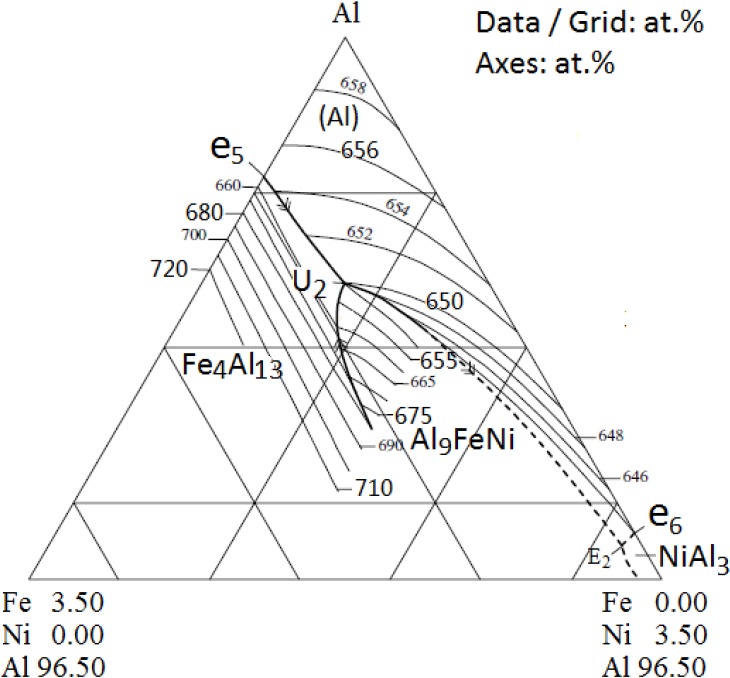
Al–Fe–Ni partial liquidus projection of Al-corner [19].

From Figure 1 it could be seen that under the condition of slow cooling, phase Al_9_FeNi was the predominant intermetallic in the as-cast microstructure in the studied alloy with 1.10% iron and 1.03% nickel. According to the Al–Fe–Ni phase diagram [19], shown in Figure 9, alloy Al–1.10Fe–1.03Ni would start to solidify with the precipitation of primary Al_9_FeNi at about 938 K (665 °C); then eutectic reaction L → Fe_4_Al_13_ + Al_9_FeNi follows; finally peritectic reaction L + Fe_4_Al_13_ → α-Al + Al_9_FeNi would occur at around 923 K (650 °C). But this investigation on the solidification of the studied alloy indicated that the solidification temperature of the alloy was about 912 K (639 °C) and the primary phase was α-Al, instead of Al_9_FeNi. The actual solidification temperature is about 26 K (26 °C) lower than that predicted by the Al–Fe–Ni phase diagram. Therefore, it could be concluded that the presence of copper and magnesium affected the solidification behavior of the Al–Fe–Ni system.

With the decrease in temperature of the molten aluminum to its solidification temperature, the primary α-Al precipitated and formed the matrix of the material. In aluminum alloy, the solute iron possessed strong tendency of segregation and formed iron containing intermetallic compounds [20]. During solidification, solutes segregated to the interface front of solidification. The solutes iron and nickel reached the eutectic composition first in the interface front, and then eutectic reaction L → α-Al + Al_9_FeNi occurred. The interrupted water-quenching test indicated that the solid fraction in the quenched sample that took place 16 min after solidification was about 28%. The eutectic reaction L → α-Al + Al_9_FeNi took place at the early stage of the solidification process. Many growing grains did not meet each other; the liquid had not yet been separated by the growing gains. In those areas, there was enough room for the Al_9_FeNi phase to grow into a radiated type structure. With the decrease in temperature, the solid fraction increased. More and more growing grains met each other; the liquid had been gradually separated by the growing grains and limited to the triangle grain boundaries. This was confirmed by the fifth interrupted water-quenching test (20 min after solidification at about 901 K (628 °C)). The Al_9_FeNi formed at lower temperature would not have enough room to extend; so it grew as flower-like morphology within the triangle grain boundaries.

With the solidification continuing, the remaining liquid at inter-grain and interdendritic regions was enriched with solutes; the eutectic reaction L → α-Al + Al_7_Cu_4_Ni followed. According to the Al–Cu–Ni diagram [17], the eutectic reaction L → α-Al + Al_7_Cu_4_Ni occurred between 858 and 808 K (585–535 °C). The precipitation temperature of Al_7_(CuNi)_5_ in this study was close to this range. Then, the eutectic reaction L → α-Al + Si took place. In the final solidification zone, the liquid was enriched with copper and magnesium and formed two intermetallic compounds Al_2_Cu and Al_2_CuMg.

There were two kinds of Al_2_Cu. One formed in the binary eutectic reaction L → α-Al + Al_2_Cu. When the eutectic composition was reached at the interface front, the eutectic reaction occurred and eutectic compounds formed and appeared as blocks. The formation of Al_2_Cu reduced the concentration of copper in the remaining liquid, which created the binary eutectic reaction L → α-Al + Al_2_CuMg. The intermetallic compound Al_2_CuMg formed lamellar or Chinese script morphologies, which were the typical eutectic structure. The other kind of Al_2_Cu formed in the ternary eutectic reaction L → α-Al + Al_2_Cu + Al_2_CuMg. Both Al_2_Cu and Al_2_CuMg appeared as blocky particles. The solidification process ended with the ternary eutectic reaction.

When the cooling rate was increased to 1.47 K·s^−1^ with Sample AC, the solidification rate greatly increased. Once the solidification started with the formation of the primary α-Al, the liquid was quickly separated by the growing α-Al. Therefore, in this sample, no radiated type structure could form; the eutectic reaction L → α-Al + Al_9_FeNi was limited in the regions isolated by the growing α-Al. Only flower-like structures were observed in the triangle grain boundaries.

### 3.2. The As-Microstructure Characteristics of the Alloy under Near-Rapid Cooling

**Figure 10:**
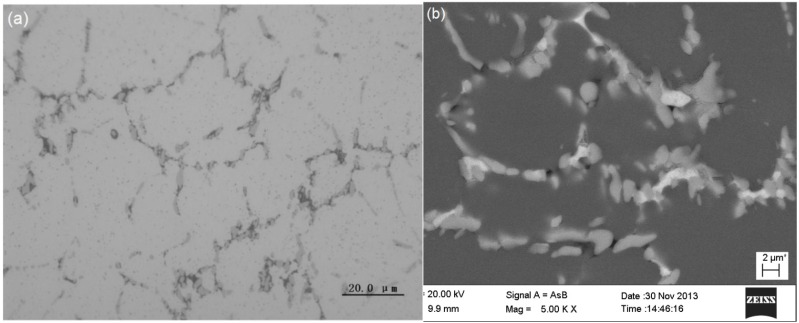
As-cast microstructures of the Sample IC: (**a**) OM image; (**b**) SEM backscatted electron image.

When the alloy solidified at a higher cooling rate, significant change took place in the as-cast microstructure. Figure 10 shows the as-cast microstructure of Sample IC, solidified at a cooling rate of 39.5 K·s^−1^. No flower-like eutectic cluster and fine eutectic cellular were observed. Only some eutectic structure formed at grain boundaries. At high magnification, it was observed that the eutectic structure consisted of two intermetallic compounds. The EDX and EPMA analyses, as shown in Table 4, indicated that the white phase contained mainly aluminum and copper, while the gray phase contained mainly aluminum, iron and nickel. But the contents of copper in the white phase and the iron and nickel in the gray phase were relatively low; much lower than the stoichiometric compositions of the equilibrium phases. It was assumed that they were metastable states. The white phase could be the metastable state of Al_2_Cu, while the gray phase could be the metastable state of Al_9_FeNi. The eutectic structures were the product of ternary eutectic reaction of L → α-Al + metastable Al_2_Cu + metastable Al_9_FeNi. However, further detailed research is needed to study the crystal structures and properties of those phases formed under the condition of near-rapid solidification with lower chemical compositions.

**Figure 11:**
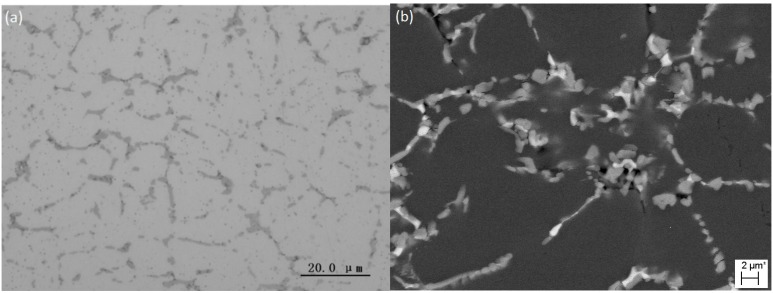
As-cast microstructures of the Sample WC: (**a**) OM image; (**b**) SEM backscatted electron image.

Increasing cooling rate from 39.5 to 88.2 K·s^−1^ did not change the properties of the as-cast microstructure of the alloy. Figure 11 shows the as-cast microstructure of Sample WC solidified at a cooling rate of 88.2 K·s^−1^. It was observed that the morphologies and the distribution of the intermetallic compounds were almost the same as those observed in the previous sample; but the sizes of the grains and the compounds were further reduced and the amount of white phases increased. The eutectic structures were also composed of two intermetallic compounds and they were also metastable states. The EDX and EPMA analyses, as listed in Table 5, indicated that the compositions of the phases were close to those observed in Sample IC. Therefore, the white phase could also be the metastable state of Al_2_Cu, while the gray phase could also be the metastable state of Al_9_FeNi.

**Table 4:** The compositions of intermetallic compounds in Sample IC measured by EDX and EPMA (at%).

Phase	EDX/EPMA	Al	Cu	Mg	Fe	Ni	Suggested Phase formula
White	EDX	78.76	18.04	1.92	0.53	0.76	Metastable Al_2_Cu
	EPMA	75.44	16.40	1.28	2.83	3.85	
Gray	EDX	85.37	2.23	1.43	5.4	5.49	Metastable Al_9_FeNi
	EPMA	79.67	3.92	3.27	6.04	6.70	

**Table 5:** The compositions of intermetallic compounds in Sample WC measured by EDX and EPMA (at%).

Phase	EDX/EPMA	Al	Cu	Mg	Fe	Ni	Suggested Phase formula
White	EDX	77.81	18.18	2.03	0.77	1.20	Metastable Al_2_Cu
	EPMA	66.91	24.38	1.03	2.83	4.51	
Gray	EDX	85.02	3.55	1.18	5.11	5.13	Metastable Al_9_FeNi
	EPMA	84.65	1.13	1.09	6.50	6.32	

The cooling rate had great influence on the as-cast microstructure of the alloy. It was interesting and necessary to analyze the solidification process of the alloy.

In Sample IC, which was solidified at a cooling rate of 39.5 K·s^−1^, the as-cast microstructures were quite simple with only primary α-Al plus metastable intermetallics Al_9_FeNi and Al_2_Cu, which could be attributed to the high cooling rate. Under the condition of near-rapid solidification, the solute segregation was limited by two factors. One was that the grain size and arm space of the dendrite was reduced, which gave rise to the increase in the number of final solidification zones in the grain boundaries and inter-dendrite regions. In return, the concentration of solutes in the final solidification zones was thinned. The other factor was that the fast solidification speed limited the enrichment of solutes in the final solidification zone, since the rejection of solute into the interface front was limited. Therefore, when the final solidification temperature was reached, the concentration of the solutes was not able to reach the equilibrium composition of eutectic compounds, resulting in products from the eutectic reaction being metastable phases.

Cooling rate is of great influence on the solidification and segregation behavior of the alloy. Zhang and Gao [21] conducted an investigation on the microstructure of rapid solidified Al–4Cu–Mg–3Fe–4Ni alloy and found that under the condition of rapid solidification, only intermetallic Al_3_Ni was observed in the as-spun sample; almost all the solute atoms were trapped in the α-Al matrix. Therefore, the formation of coarse intermetallics could be suppressed by fast cooling. This study concluded that increasing cooling rate from slow cooling to near-rapid cooling, the as-cast microstructures changed from complex multi eutectic compounds to simple metastable ternary eutectic compounds.

The as-cast microstructures can be simplified by near-rapid solidification. The coarse intermetallics formed under slow cooling can be avoided by increasing the cooling rate. In other words, the intermetallics can be refined to a great extent by near-rapid cooling. This is very interesting to the development of this kind of alloy. It is assumed that under near-rapid solidification, such as in the process of continuous strip casting, the alloying elements can be increased to a high level without forming coarse intermetallic compounds, which will increase the precipitation of strengthening phases. Hence, higher strength can be achieved.

## 4. Conclusions

Under the condition of slow cooling, AA2618 alloy was of complex as-cast microstructures with lots of eutectic compounds including Al_9_FeNi, Al_7_(CuNi)_5_, Si, Al_2_Cu and Al_2_CuMg. The intermetallic compound Al_9_FeNi was very developed with both flower-like and radiated type structures. The radiated type structure extended through several grains.

Phase Al_9_FeNi was the dominant intermetallic compound, which precipitated at the earlier stage of solidification by eutectic reaction L → α-Al + Al_9_FeNi, and grew into radiated type structure. As the alloy continued to solidify and the solid fraction increased, the eutectic reaction was limited within triangle grain boundaries and the intermetallic formed flower-like morphologies.

Increasing cooling rate would suppress the formation of radiated type structures of intermetallic Al_9_FeNi. The eutectic cluster of Al_9_FeNi mainly displayed flower-like. The eutectic cellular increased in quantity.

Under the condition of near-rapid cooling, the as-cast microstructure of the alloy consisted of primary α-Al plus metastable intermetallics Al_9_FeNi and Al_2_Cu; the equilibrium eutectic compounds were suppressed.

The intermetallics could be refined to a great extent by near-rapid cooling.
